# Minimising Tourniquet Time and Post-operative Pain During Carpal Tunnel Decompression

**DOI:** 10.7759/cureus.5146

**Published:** 2019-07-16

**Authors:** Rafia Ghani, James E Archer, Munawar Shah

**Affiliations:** 1 Trauma & Orthopaedics, Walsall Manor Hospital, Walsall, GBR

**Keywords:** surgical, orthopaedics, upper extremity, wrist, tourniquets, pain, carpal tunnel, complications

## Abstract

Background

Pain and discomfort at the tourniquet and wound site are recognised complications of carpal tunnel surgery. Studies have shown that longer tourniquet times lead to increased pain, local and systematic complications. We hypothesise that minimising the intraoperative tourniquet time will reduce post-operative pain and complications.

Objective

Our aim is to present the results of our novel operative technique for carpal tunnel decompression which minimises tourniquet time.

Method

The study represented a prospective case series in which 55 consecutive and unselected patients with positive nerve conduction study results were operated on by a single surgeon at a single hospital site over a period of 12 months. The patients filled in a questionnaire based on a visual analogue score (VAS) (1-10) for pain at (1) first presentation at the clinic, (2) recovery in theatre post-operatively and (3) on discharge from care at 12 weeks post-operatively. Questions included the perception of pain at the tourniquet site and at the wound site.

Results

A total of 55 (female 39 and male 16) patients participated in the study. The average tourniquet time was 5 minutes 50 seconds. 98% of patients had a VAS of 1 at both the wound and tourniquet site post-operatively. One patent had a score of 3 at the tourniquet site. Eleven patients had undergone contralateral carpal tunnel decompression surgery. Of the remaining 43 patients, all said they would have the other side operated on in the same way.

Conclusion

We have demonstrated a safe and efficient technique to reduce post-operative pain by minimising tourniquet inflation time. Our patient cohort experienced no significant complications and minimal pain post-operatively.

## Introduction

Carpal tunnel decompression under local anaesthetic is an accepted method of treatment for carpal tunnel syndrome [1]. Pain and discomfort at the tourniquet and wound site is a recognised complication of surgery [2]. A number of techniques have been described to manage pain and they have associated advantages and disadvantages. Regional anaesthesia requires specific training and is not without risk with reported fatalities with the use of intravenous (IV) regional anaesthesia [3]. Brachial plexus block has been shown to have a high learning curve and a high rate of incomplete anaesthesia [4]. A combination of local anaesthetic and tourniquet has been shown to be a safe and replicable technique for carpal tunnel decompression [5]. However, studies have shown that longer tourniquet times lead to increased pain [6-8]. Furthermore, a prolonged tourniquet time has been shown to have local and systemic complications [9]. It has been demonstrated that upper arm tourniquets can be tolerated for an average time of 18 minutes [10].

Our aim is to present a safe and reproducible operative technique for carpal tunnel decompression under local anaesthetic which aims to minimise post-operative pain caused by the tourniquet. Our technique focuses on reducing the length of tourniquet time by the surgeon to help reduce the post-operative pain for patients.

## Materials and methods

Fifty-five consecutive and unselected patients with positive nerve conduction study results were operated on by a single surgeon at a single site over a period of 12 months. The patients filled in a questionnaire based on a visual analogue score (VAS) (1-10) for pain at (1) first presentation at the clinic, (2) at recovery in theatre post-operatively and (3) on discharge from care at 12 weeks post-operatively. Questions included the perception of pain at the tourniquet site and at the wound site.

Patient pathway

The procedure is explained to all patients during the consent process in the clinic. All patients are admitted as a day case procedure.

**Figure 1:**
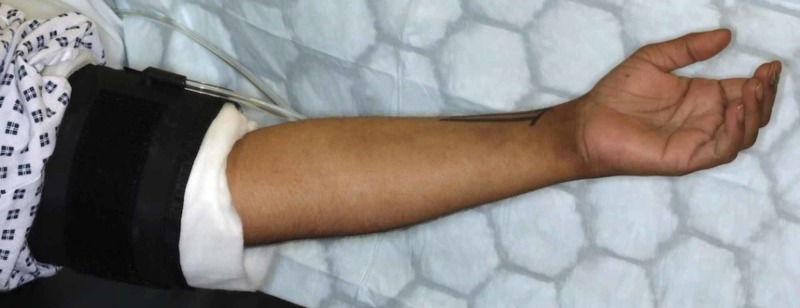
Positioning of tourniquet

**Figure 2:**
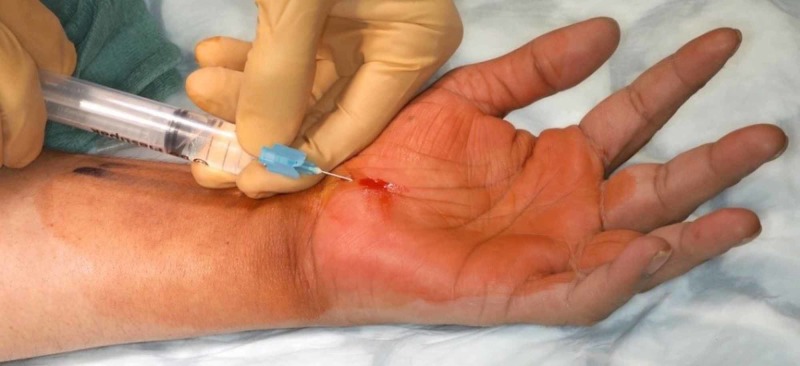
Infiltration of local anaesthetic

**Figure 3:**
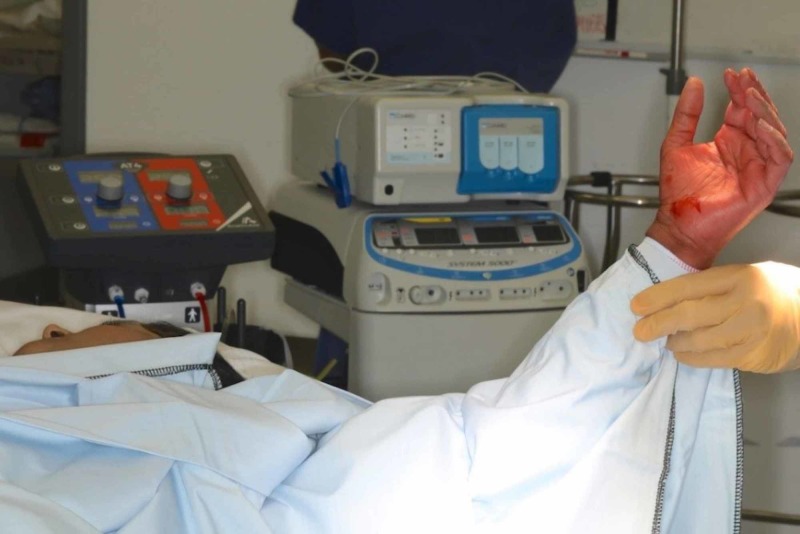
Elevation of the arm

**Figure 4:**
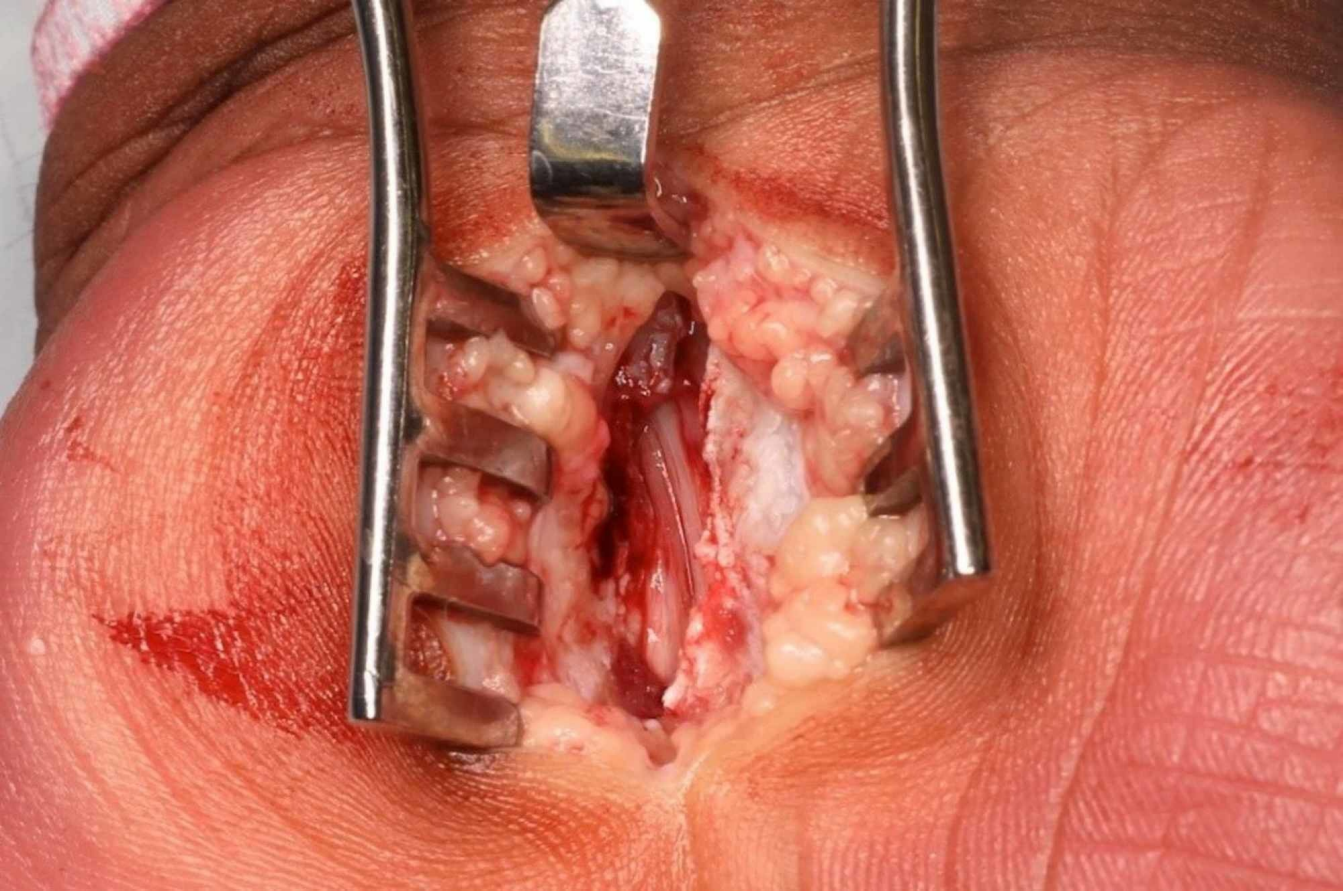
Wadsworth incision and decompression

Once in the operating theatre complex, the tourniquet is applied to the upper arm but not inflated in the anaesthetic room (Figure 1). Local anaesthetic (10 ml of 1% lignocaine and 10 ml of 0.5% bupivacaine) was infiltrated into the line of incision. Local anaesthetic (10 ml of 2% lignocaine and 10 ml of 0.5% Marcaine) was infiltrated into the carpal tunnel (Figure 2). The arm is elevated whilst the surgeon is scrubbing. The hand is prepared with alcoholic chlorhexidine with the arm held high by an assistant (Figure 3). The hand is draped in the same position and the tourniquet inflated to 100 mm above systolic blood pressure. A standard Wadsworth incision is made and the carpal tunnel is decompressed (Figure 4). The tourniquet is released at this point and any bleeding vessels are cauterised using bipolar diathermy. The wound is sutured with 3-0 nylon. The surgical site is protected with wool and crepe, and the arm is placed in a Bradford sling. The patient is seen at two weeks for removal of sutures and discharged at their final review 12 weeks post-operatively.

## Results

Fifty-five patients were included in this study and their demographics are displayed in the table below (Table 1). The average tourniquet time was 5 minutes 50 seconds. 98% of patients had a VAS of 1 at both the wound and tourniquet site post-operatively. One patient had a score of 3 at the tourniquet site. Eleven patients had undergone contralateral carpal tunnel decompression surgery. Of the remaining 43 patients, all said they would have the other side operated on in the same way.

**Table 1:** Demographic details

Characteristics	Number
Median Age years, months (range)	52,3 (26,7-91,1)
Participants, n	55 (39F: 16M)
Dominant Hand (Right Hand: Left Hand)	43:12

## Discussion

Carpal tunnel release has been shown to be an excellent procedure in relieving pre-operative pain and has a high patient satisfaction [11]. The main outcomes that matter to patients are pain and return of normal function [12-14]. There have been many attempts to modify the technique to achieve minimal post-operative pain and we feel we have developed a safe and replicable technique to achieve this. 98% of our cohort had the lowest score possible for pain post-operatively at both the tourniquet and operative site. By carefully timing inflation and deflation of the tourniquet on decompression of the carpal tunnel, we were able to achieve an average tourniquet time of 5.5 minutes. This is shorter than documented times for upper-arm tourniquet which was on average 9.5 minutes [15]. 100% (43) of our patients who could have carpal tunnel decompression on the contralateral wrist agreed that they would have the procedure done in the same way, reflecting their overall satisfaction with the whole procedure. There were no other significant complications immediately post-operatively or at one-year follow-up.

The main limitation of our study is that we have described a single surgeon technique, therefore, our results may not be replicable accounting for variation in surgeon experience and skill. Secondly, patient-related outcome measures were not assessed, as our main outcome measure was post-operative pain scoring.

## Conclusions

We have demonstrated a safe and efficient technique for carpal tunnel release under local anaesthesia and tourniquet. By focusing on carefully timing the inflation and deflation of tourniquet, we were able to minimise overall tourniquet time and reduce pain for the patient. Our patient cohort experienced no significant complications and 98% of our cohort had no pain post-operatively and at follow-up.
